# Theocin-Sodium Acetate in the Relief of Dropsy

**Published:** 1908-06-06

**Authors:** 


### THEOCIN-SODIUM ACETATE IN THE RELIEF OF DROPSY.

Theobromine is a vegetable alkaloid which is, in organic chemical nomenclature, a di-methyl-xanthine. There are several different di-methyl-xanthines: theocin is the name of one which is synthetically prepared. All the members of this class of drugs have a potent diuretic action. Theocin-sodium acetate is one which has commended itself very greatly in practical use.

It is chiefly for cardiac dropsy that it is employed. Acute renal dropsy is less likely to be thus treated, because with inflamed kidneys it is generally held that active diuretics are to be avoided. The kind of cardiac dropsy seems to matter little. It may be that of valvular heart disease, of myocardial degeneration, or of failure of the right side of the heart from chronic troubles in the lungs, or from arterio-sclerosis or granular kidney.

Two points which seem to have been fully established are: first, that theocin-sodium acetate acts very much better when the patient is having a digitalis preparation at the same time, particularly if the digitalis has been already administered for three or four days before the theocin salt is added; secondly, that patients are apt to become tolerant of the drug quickly, so that it is better administered periodically rather than continuously.

It might be thought that, since the benefit of digitalis itself is seldom fully marked until the fourth or fifth day after its administration has been begun, the good that has been attributed to the theocin salt when it has been started on that day is really due to the digitalis entirely. This, however, seems not to be the case. If similar patients are treated with digitalis only, with theocin-sodium acetate only, and with the two together, respectively the best results follow the combination.

In a typical case the patient is put to bed, and 15 minims of standard tincture of digitalis are given four times a day, either in half an ounce of plain water or in a little dill water or chloroform water, but not in association with caffeine citrate, squills, nux vomica, or other similar drug.

It should be remembered that digitalis can be given in large doses without producing nausea or vomiting if it is given by itself, whereas the same doses administered with other medicines in the mixture along with it will again and again upset the stomach. Instead of the tincture, some authorities prefer to give the fresh infusion; it matters little which is used, provided that the chemist has ascertained that the preparation supplied is physiologically active.

When the digitalis has been continued in this way for three days theocin-sodium acetate is prescribed in 6-grain doses thrice daily. It is often added to the digitalis mixture, but if there is the slightest sign of nausea it is much better to give the two at separate times, each in a simple excipient. Doses of 6 grains are usually sufficient, though 10 grains may be prescribed if necessary. The daily volume of urine voided will usually go up almost immediately, and within a fortnight the dropsy should have decreased very much. The theocin salt may or may not be omitted after this, digitalis being continued for a longer time according to the nature and the progress of the case.

